# Bibliography

**Published:** 1894-04

**Authors:** 


### Bibliography.

Catching's Compendium of Practical Dentistry for 1893. B. H. Catching, D.D.S., Editor and Publisher. Atlanta, Ga., 1894.

This compendium of practical dentistry is presented to the dental reading public with a promptness to be commended. It contains, as heretofore, abstracts from all the most valuable papers presented on this side of the ocean, with a few from other countries.

This is a marked improvement over previous issues, and we are pleased to notice the editor appreciated the suggestion made in regard to this in the review made by this journal of the issue for 1892. The editor promises an enlarged outlook for that of 1894, for he says in his preface, "In this issue the condensed practical results of the dental journals of six different nations are given. The next (1894) edition will contain the practical results of the dental journals of the world. An editor and translator for each language in which a journal is published will give, in condensed style, the work of each country. . . . The proposed cosmopolitan character of the work is begun in this issue by the assistance of able editors and translators of the German, French, and Italian. W. D. Miller, M.D., D.D.S., of Berlin, A. C. Hugenschmidt, M.D., D.D.S., of Paris, and Wm. Dunn, D.D.S., of Florence," will act in this capacity.

The amount of labor required to make a book of this kind satisfactory both to writers and readers can hardly be appreciated by those not familiar with the work of selecting and condensing from the immense amount of material yearly put forth. While in many respects the subject matter is not of the highest scientific order, it is an excellent *résumé* of the work of the year. The fault, if fault it be, lies not with the editor, but with writers on dental topics. There is too much of an effort in all the proceedings of dental societies to make a lengthy essay, without much regard to scientific accuracy of statement. Perhaps there is no better way of exhibiting this than a book of this kind. It enables us to gauge our shortcomings. It has, however, a higher object, and this makes it especially valuable to the busy dentist, in that he has a synopsis of the yearly work for ready reference. In this respect this compendium becomes a necessity in the operating-room, laboratory, and at the desk.

It is to be regretted that the editor has taken the responsibility of changing the titles of some of the papers from which he has taken abstracts. Especially is this noticeable in those on pyorrhœa alveolaris. It is no improvement to head all these "Riggs's Disease." The dental profession has long since condemned the use of this name, and it should not appear as descriptive of a pathological condition at this stage of the investigation of this difficult subject.

The editor has performed an excellent work in giving this compendium to the profession, and the extension world-wide will add not only to its value, but to its interest. It is a satisfaction to know that it "has passed the experimental stage," and it is to be hoped the demand for it will grow with each succeeding year.

Popular Essays upon the Care of the Teeth and Mouth. By Victor C. Bell, A.B., D.D.S., Director of the Special Prosthetic Department of the New York College of Dentistry, Late Dental Surgeon to the German Polyclinic. Published by the Author, 1894.

Perhaps one of the greatest difficulties met with in dentistry has been the ignorance of the general public, both in regard to personal care of teeth as well as the value of dental services. A half-century ago this was the chief stumbling-block in the practice of that period, for at that time, the majority of persons had no higher conception of a dentist's duty than as a "puller of teeth," and it is therefore not surprising that dental services then were mainly confined to this.

Since that period a great change has been effected, but so much is still to be desired in this direction that any effort made to enlighten the general public must be received with pleasure. Hence Dr. Bell's effort is one of the most satisfactory of any in this direction.

He has stated in his preface a great truth, when he says, "Were the information contained in this little book generally diffused, and its teachings well followed, not only would very much pain and suffering be prevented, but the general term of human life would be perceptibly lengthened."

The author devotes the one hundred and two pages of the book to general instruction, arranged in a series of essays, free from technicalities and well adapted to popular comprehension.

There are necessarily many difficulties in the preparation of such a volume, and the author has probably found this to be true in many of the essays. It is a question whether he had not better have omitted altogether "Hints on Home Remedies," not but that it contains excellent suggestions, but that it is questionable whether patients are, as a rule, capable of carrying these out. For an untrained person to make a correct diagnosis of toothache, in its protean forms, seems to be an impossibility. In illustration of this, in describing the treatment of "Aching Teeth when the Pulp is Dead," the author gives this advice: "Rub iodine and aconite, in equal parts, around the gums with cotton or a camel's-hair brush. . . . The iodine and aconite induce a healthy flow of the blood, and facilitate the removal of waste material." Aside from the difficulty of the patient comprehending when this should be used, or to differentiate between pericementitis and pulpitis, there is a wrong committed in advising the patient to use an agent as dangerous as aconite. This should never go out of the office, and it requires a full comprehension of its toxic properties to be used there. Aside from this, it is, in the opinion of the reviewer, a useless remedy when applied topically to reduce inflammation, as it paralyzes the sensory nerves and produces torpidity where the greatest amount of activity is required, if counter-irritation is to be of any avail in reducing the congestion at the focus of inflammation.

The author still adheres to the idea that lime-water taken internally will harden teeth. It seems useless to contend this point, but assimilation and nutrition do not proceed from inorganic material.

The book, as a whole, can be cordially commended, and if it could be placed, in a cheap form, in dentists' hands, it would unquestionably be productive of a vast amount of good. It is not probable, unless given away, it will reach to any large extent those for whom it is written, as the general public are not yet sufficiently well educated, it is feared, to appreciate the importance of dental subjects by investing in this character of literature.

